# Neural correlates of semantic typicality during episodic memory retrieval in autism spectrum disorder

**DOI:** 10.1038/s41598-025-19086-4

**Published:** 2025-09-17

**Authors:** Ann-Kathrin Beck, Cristiane Souza, Margarida V. Garrido, J. Bernardo Barahona‑Correa, Joana C. Carmo, Thomas Lachmann, Daniela Czernochowski

**Affiliations:** 1 University Kaiserslautern-Landau RPTU, Kaiserslautern, Germany; 2 Iscte - Instituto Universitário de Lisboa, Lisbon, Portugal; 3 Champalimaud Clinical Centre, Champalimaud Centre for the Unknown, Lisbon, Portugal; 4 NOVA Medical School, Universidade Nova de Lisboa, Lisbon, Portugal; 5 CADIn – Neurodesenvolvimento e Inclusão, Cascais, Portugal; 6 Lusófona University, Digital Human-Environment Interaction Labs, HEI‐Lab, Lisbon, Portugal; 7 Centro de Investigación Nebrija en Cognición (CINC), Universidad Nebrija, Madrid, Spain

**Keywords:** Psychology, Medical research

## Abstract

This study examined the effects of item typicality (typical vs. atypical), encoding type (categorical vs. perceptual), and neurodivergence (autistic vs. neurotypical male adults) on memory discrimination and associated neuronal patterns. Despite similar overall memory discrimination performance between groups, analyses of event-related potentials revealed that neurotypicals displayed an early ERP effect, suggesting reliance on familiarity-driven processes. In contrast, autistic participants showed a later ERP modulation, indicating a reliance on recollection-based processes. Notably, relying on either familiarity or recollection influenced the activation in the post-old/new-response period, in which only neurotypical adults needed to reinstate item details for the subsequent Remember-Know-Guess (R-K-G) judgments. These findings suggest that autistic adults may recruit different cognitive processes to achieve memory performance comparable to neurotypical adults. Additionally, our results suggest that item typicality interacts with encoding type in modulating the cognitive processes underlying memory retrieval and their neural correlates in both autistic and neurotypical adults. The study highlights the need to investigate the role of semantic processes in episodic memory retrieval in both neurotypical and autistic individuals.

## Introduction

When retrieving information from memory, the interaction between semantic and episodic memory systems plays a crucial role^1^. While episodic memory is tied to personal experiences and allows individuals to re-experience past events in a context-rich manner (e.g., the last time you ate an apple), semantic memory is recruited when retrieving context-free factual knowledge organized by content type (e.g., that apples are fruits) rather than by specific details regarding occurrence. There is consistent empirical evidence that these two types of memory are both structurally and functionally dissociable^2^. According to the dual-process model of episodic memory, recognition memory relies on two distinct processes: familiarity and recollection^2,3^. Familiarity provides a general, context-free sense of recognition (i.e., I know I have heard this song somewhere before). In contrast, recollection involves a more deliberate search for contextual details and hence can support the retrieval of source-specifying context features (i.e., I heard this song yesterday on the radio and the lyrics reminded me of calling a friend) and relies on the hippocampus^3^. Compared to familiarity-based retrieval, recollection is generally slower, more effortful, and associated with higher confidence. However, the two processes can only be reliably disentangled by using self-reported Remember-Know-Guess (R-K-G) judgements or the temporo-spatial characteristics of event-related potentials (ERPs)^4^. When participants are asked to identify items they have encountered in a previous study phase, stimuli correctly identified as old are associated with more positive amplitudes compared to stimuli correctly identified as new (i.e., old/new effect). Familiarity-based memory retrieval (i.e., a Know response) is usually indexed by an early frontal old/new effect approximately 300–500 ms after stimulus onset, whereas recollection (i.e., a Remember response) is indexed by a parietal old/new effect around 500–800 ms^5^. The present study examines how these two processes are influenced by item typicality of the study material, by the type of processing during initial encoding (i.e., conceptual or perceptual encoding task), and neurodiversity status (i.e., autism spectrum disorder; ASD). While few neuroscientific studies explicitly focus on the interaction between episodic and semantic systems, these interactions may be particularly relevant for understanding cognitive processes in ASD, given recent evidence suggesting atypicality in semantic categorization^6^. Moreover, in most behavioural and neuroscientific studies, typical and atypical items are not directly compared (as salience is associated with better memory performance for unexpected information^7^). Recent findings in each of these areas will be described below.

There is ample evidence that conceptual knowledge has a decisive impact on semantic categorization and declarative memory^7–9^. This has traditionally been demonstrated by manipulating item typicality. Typical items closely match the prototypical representation stored in semantic memory (e.g., sparrow as a bird), whereas atypical items deviate from these prototypes and share more attributes with other categories^10–12^ (e.g., ostrich as a bird). Alves and Raposo found that typical items were associated with increased familiarity-based responses (e.g., I believe I have seen a sparrow somewhere lately), whereas atypical items enhanced overall recognition and recollection-based responses^13^ (e.g., the last time I saw an ostrich was in the spring with my friends at the zoo). Activating conceptual knowledge during initial item encoding also improves subsequent retrieval, such as the ability to distinguish old from new items, even when perceptual features are slightly changed between study and test^14,15^. Furthermore, Souza et al. showed that categorical encoding (e.g., To what category does it belong?) is associated with subsequent increased familiarity, while perceptual encoding (e.g., How complex is this picture?) led to increased recollection at testing^16^. They also found that following categorical encoding – but not perceptual encoding – recollection was lower for typical items than for atypical ones, suggesting that presenting typical items during categorical encoding diminishes during memory reinstatement.

While in ASD semantic memory remains mostly intact (e.g.^6,17–19^), there is evidence that ASD individuals tend to self-report fewer recollective experiences (^20^for a review), despite similar old/new discrimination^19,21^. More recent evidence has shown that autistic participants take longer to classify objects into semantic categories and base their decision**s** on arbitrary item features, as evident in modulations of the ERP component P3 (elicited during categorization based on arbitrary features^22^). This is in contrast to NT adults, who base their ultra-rapid semantic categorization on simpler object features that are assessed in the time range of the ERP component P2^23^ (observed when targets are discriminated based on simple features^24^). When comparing autistic and NT adults in terms of item typicality and encoding type, autistic adults were less accurate and slower than NT adults and, unlike NT participants, did not show better recognition for atypical items^25^. In line with previous findings, autistic participants provided fewer recollection responses, which could be related to difficulties in predicting category membership at encoding or modulating retrieval strategies accordingly. Complementing these behavioural results, in ASD, Massand and Bowler observed an attenuated early frontal old/new effect^26^ associated with familiarity, suggesting qualitative differences in memory retrieval mechanisms.

Since ASD is known to be associated with differences in attention allocation, predictive processing, and communication, we hypothesize that autistic individuals may access conceptual and episodic information differently when retrieving memories. In this study, we assess whether the typicality of the material, the cognitive processes involved during initial encoding, and neurodivergence can influence later memory performance as well as the memory systems engaged during information reinstatement. We propose that autistic participants may not modulate memory retrieval processes as a function of the processes involved during initial encoding. To this end, we analyze ERP data collected alongside the behavioral data (i.e., response times during encoding, recognition accuracy, and response times, as well as self-reported R-K-G judgment probability and reaction times) reported in Souza et al.^25^ to investigate whether retrieval processes differ qualitatively or quantitatively as a function of item typicality (typical vs. atypical), encoding type (categorical vs. perceptual), or neurodivergence (autistic vs. NT adults). By integrating novel behavioural analyses of false alarms and response bias, and complementing them with the ERP components analysis, we aim to determine whether autistic adults show differences in the modulation of retrieval processes based on prior encoding conditions. For the behavioral indices, we computed a repeated-measures ANOVA on the within-subject factors Encoding Type (categorical vs. perceptual) and Item Typicality (typical vs. atypical), as well as the between-subject factor Neurodivergence Group (ASD vs. NT). Regarding the ERP results, based on the findings by Beck et al.^23^, who found qualitatively different neuronal processes underlying categorization (specifically in relation to item typicality) when comparing autistic and NT adults, we also explored whether both our experimental factors modulate the neuronal processes during recognition memory retrieval. To investigate these modulations, we used the time windows commonly used to assess old/new effects in NT participants (see, e.g.^5^). Note that standard EEG paradigms focus only on typical items and often require participants to retrieve contextual features along with the old/new response. Since we were specifically interested in the role of conceptual knowledge in modulating episodic memory retrieval, we did not restrict our analyses to compare old and new items but used planned contrasts to assess the effects of item typicality and encoding type as well. To this end, we performed one-way repeated measure ANOVAs with the within-subject factor Condition (Categorical typical, Categorical atypical, Perceptual typical, Perceptual atypical, and New) on the mean amplitude for the respective time windows. To compare old and new items, we conducted planned contrasts between all old conditions and new items. To assess the effects of item typicality and encoding type, we conducted planned contrasts between categorical and perceptual encoding tasks as well as between typical and atypical items. Reliable interactions were followed up with specific contrasts (i.e., categorical typical vs. atypical, perceptual typical vs. atypical, typical categorical vs. perceptual, atypical categorical vs. perceptual).

## Results

### Memory performance

Overall, both autistic (*M* = 0.59, *SE* = 0.03) and NT (*M* = 0.65, *SE* = 0.03) participants exhibited a high memory discrimination (sensitivity score Pr^27^). As summarized in Table 1, in every condition discrimination was only slightly higher in NT participants (*p* =.18). The repeated-measure ANOVA for Pr revealed a main effect of Item Typicality, *F*(1,28) = 14.6, *p* <.05, *η*^2^_p_ = 0.34, with higher discrimination for atypical (*M* = 0.64, *SE* = 0.02) than typical (*M* = 0.60, *SE* = 0.03) items. Additionally, we observed a main effect of Encoding Type, *F*(1,28) = 7.6, *p* <.05, *η*^2^_p_ = 0.21, with higher discrimination for categorical (*M* = 0.65, *SE* = 0.02) than for perceptual (*M* = 0.58, *SE* = 0.03) encoding. These two main effects were qualified by an interaction between Encoding Type and Item Typicality, *F*(1,28) = 6.1, *p* <.05, *η*^2^_p_ = 0.18. The post-hoc analyses showed that the effect of Item Typicality was only observed after categorical (*p* <.05), but not perceptual (*p* =.43) encoding.

**Table 1:** Memory discrimination performance with mean discrimination (Pr) and response bias (Br) index values **(*SE*)**.

Group		Categorical typical	Categorical atypical	Perceptual typical	Perceptual atypical
NT	Pr	0.65 (0.04)	0.72 (0.03)	0.59 (0.03)	0.62 (0.03)
	Br	0.59 (0.06)	0.70 (0.05)	0.50 (0.06)	0.54 (0.06)
ASD	Pr	0.59 (0.03)	0.65 (0.04)	0.56 (0.04)	0.55 (0.04)
	Br	0.52 (0.07)	0.59 (0.07)	0.45 (0.05)	0.45 (0.05)

The repeated-measure ANOVA for the response bias index Br revealed no (interaction) effects with Group, even though NT adults’ responses (*M* = 0.58, *SE* = 0.05) were slightly more liberal than those of autistic adults (*M* = 0.50, *SE* = 0.06; see Table 1). We also observed a main effect of Item Typicality, *F*(1,28) = 15.7, *p* <.05, *η*^2^_p_ = 0.36, with a more liberal bias for atypical (*M* = 0.57, *SE* = 0.04) than typical (*M* = 0.52, *SE* = 0.04) items. Additionally, we observed a main effect of Encoding Type, *F*(1,28) = 13.3, *p* <.05, *η*^2^_p_ = 0.32, with a more liberal bias following categorical (*M* = 0.60, *SE* = 0.04) than perceptual (*M* = 0.48, *SE* = 0.04) encoding. Lastly, these two main effects were qualified by an interaction between Encoding Type and Item Typicality, *F*(1,28) = 8.5, *p* <.05, *η*^2^_p_ = 0.23. Post-hoc analyses showed that the effect of Item Typicality was only observed for categorical (*p* <.05), but not perceptual (*p* =.42) encoding.

## Event-related potentials

**Fig. 1:**
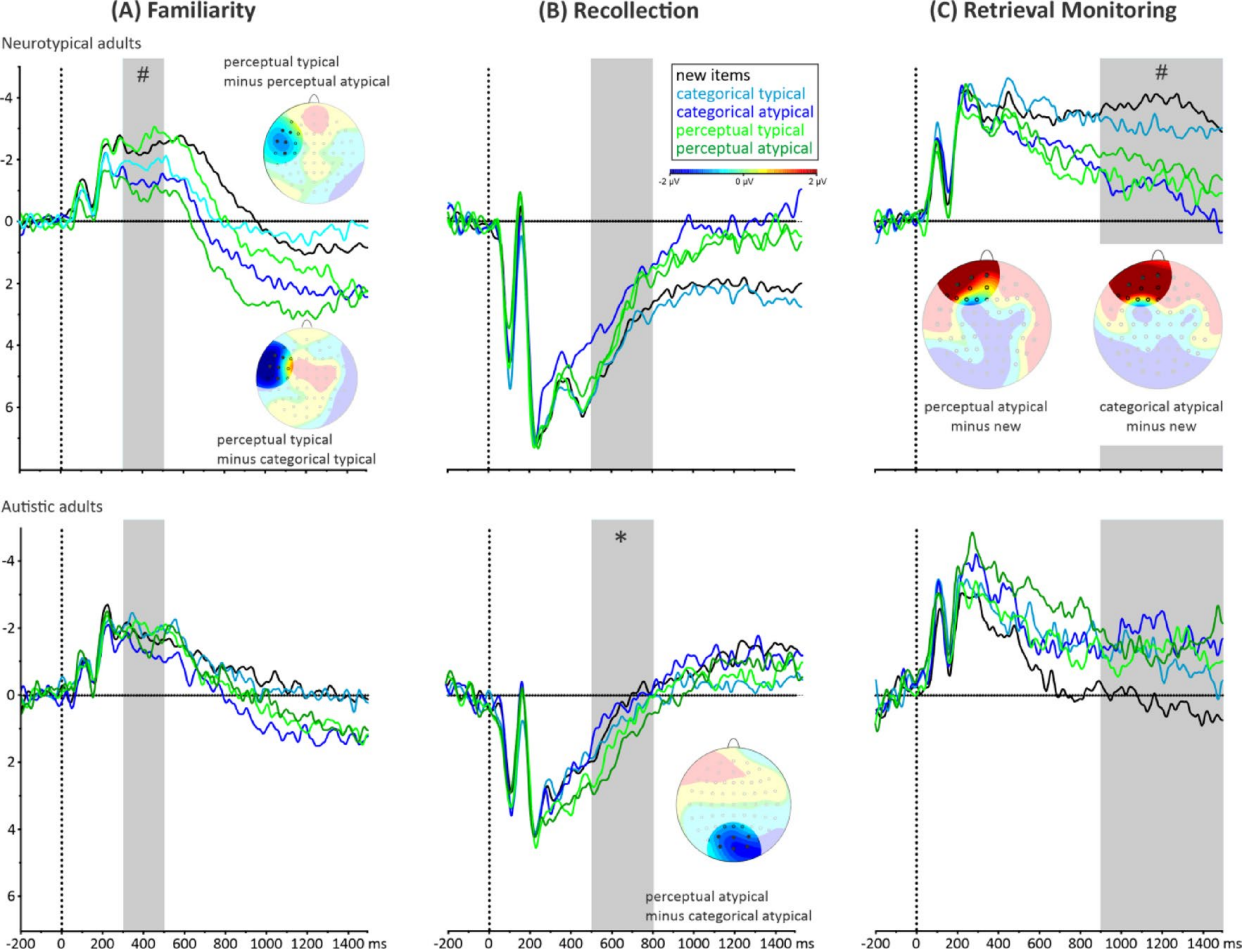
ERP waves for NT (top) and autistic adults (bottom). Three time windows were evaluated: (**A**) 300–500 ms, representing Familiarity (left), (**B**) 500–800 ms, representing Recollection (middle), and (**C**) 900–1500 ms, representing Retrieval monitoring (right). Topographical maps show the mean voltage difference for significant specified contrasts (i.e., between condition differences) within the respective time windows, at the selected electrode sites (black dots): (**A**) F7, F5, FT7, FC5, and C5 (for Familiarity), (**B**) POz, PO4, PO3, Oz, O1 and O2 (for Recollection), and (**C**) FPz, FP1, and AF7 (for Retrieval monitoring in the post-old/new-response period). Statistical significance levels for the main effect of Condition in the respective time window are indicated with an asterisk * for *p* <.05 or a hashtag ^*#*^ for *p* <.10.

Results are reported separately for NT and autistic adults, as illustrated at the top and bottom of Fig. 1, respectively.

In NT adults **(**Fig. 1, top), Condition effects on mean voltages (see Table 2) were observed only between 300 and 500 ms (i.e., the time window associated with familiarity-based memory retrieval, see Fig. 1A) and between 900 and 1500 ms (i.e., the time window associated with retrieval monitoring, see Fig. 1C). We did not observe any condition effects between 500 and 800 ms (i.e., the time window associated with recollection-based memory retrieval; *p* =.27). In the 300**–**500 ms time window, we observed a trend for the main effect of Condition, *F*(4,64) = 3.1, *p* =.08, *η*^2^_p_ = 0.16. As illustrated in Fig. 1A, a difference between typical and atypical items (*p* <.05) was observed after perceptual, but not categorical encoding (*p* =.36). Additionally, for typical items we found an effect of Encoding Type, with a more positive amplitude after categorical compared to perceptual encoding (*p* <.05). In the last time window, we observed a trend for the main effect of Condition, *F*(4,64) = 2.9, *p* =.08, *η*^2^_p_ = 0.15. As illustrated in Fig. 1C, planned contrasts revealed that categorically encoded atypical items were associated with more positive waveforms as compared to new items (*p* =.05). We also observed an old/new effect for perceptually encoded atypical items, with perceptual**ly** atypical old items having a more positive amplitude than new items (*p* <.05).

**Table 2:** Mean amplitude (and *SE*) for each time window and participant group.

	Categorical typical	Categorical atypical	Perceptual typical	Perceptual atypical	New
Familiarity (300–500 ms)	ASD: −2.0 (0.8) NT: −1.9 (0.5)	ASD: −1.3 (0.7) NT: −1.3 (0.6)	ASD: −2.0 (0.8) NT: −2.6 (0.5)	ASD: 1.6 (0.8) NT: −1.0 (0.5)	ASD: −1.8 (0.8) NT: −2.3 (0.5)
Recollection (500–800 ms)	ASD: 0.8 (1.2) NT:3.8 (1.1)	ASD: 0.3 (1.2) NT: 2.4 (1.4)	ASD: 1.3 (1.1) NT: 3.1 (1.1)	ASD: 1.6 (1.2) NT: 3.1 (1.3)	ASD: 0.4 (1.1) NT: 3.7 (1.1)
Retrieval Monitoring (900–1500 ms)	ASD: −1.0 (1.5) NT: −3.0 (1.4)	ASD: −1.8 (1.5) NT: −0.8 (1.1)	ASD: −1.2 (1.8) NT: −1.1 (0.8)	ASD: −1.5 (2.0) NT: −1.8 (1.3)	ASD: 0.3 (1.9) NT: −3.7 (1.6)

In autistic adults (Fig. 1, bottom), a Condition effect was observed only between 500 and 800 ms (i.e., in the time window associated with recollection-based memory retrieval); *F*(4,48) = 2.8, *p* <.05, *η*^2^_p_ = 0.19. As illustrated in Fig. 1B, planned contrasts revealed a difference between atypical and new items following perceptual encoding (*p* <.05). In addition, planned contrasts revealed that perceptual encoding was associated with a more positive amplitude compared to categorical encoding (*p* <.05). Moreover, we observed a difference in atypical items as a function of encoding type, with more positive amplitude after perceptual than categorical encoding (*p* <.05).

## Discussion

The present study investigated the interplay between effects of item typicality (Typical vs. Atypical), encoding type (Categorical vs. Perceptual), and the presence of neurodivergence (Autistic vs. NT male adults) on old/new memory discrimination and the corresponding electrophysiological correlates. Both autistic and NT participants displayed high old/new memory discrimination. ERP analyses revealed distinct patterns: NT participants showed an early ERP modulation of item typicality after perceptual encoding only, as well as for typical items as a function of the encoding task. In contrast, autistic individuals showed a corresponding later ERP modulation as a function of the encoding task, but this time only for atypical items. The differences in the temporal dynamics of our ERP findings align with the work reported by Souza et al.^25^, in which considerably longer reaction times were observed in this autistic sample. These findings also mirror previous results during ultra-rapid semantic categorization, in which qualitatively different modulations with a later temporal onset were observed in autistic adults^23^. Overall, our results suggest that item typicality interacts with encoding type in modulating the cognitive processes underlying memory retrieval and their neural correlates in both autistic and NT adults.

In NT participants, the early ERP time window associated with familiarity was modulated by conceptual knowledge, with perceptually atypical items eliciting more positive amplitudes than both categorically typical and perceptually typical items. These findings align with our behavioral findings on memory discrimination, which showed the highest accuracy for atypical items after perceptual encoding. Notably, we did not observe any ERP modulations in the time window associated with recollection for NT participants. This pattern of ERP results suggests that NT individuals rely on rapid, familiarity-driven processes when recognizing old items in this paradigm. Since participants are not required to retrieve any contextual item details, familiarity is sufficient to distinguish between old and new items, as reflected in their high memory discrimination. Thus, NT participants achieve fast old/new responses by postponing some aspects of the task until the subsequent R-K-G judgments. Consequently, since NT adults do not rely on recollection for old/new discrimination, they need to reinstate item details for the subsequent judgment after the initial old/new response. In the latest ERP time window, we observed evidence for the involvement of the anterior prefrontal cortex (aPFC)^28^, which is also implicated in the default mode network responsible for monitoring internal states^29^. This pattern aligns with studies emphasizing the role of dorsolateral PFC in monitoring memory retrieval and, more specifically, in integrating criteria to translate a continuous familiarity response into a R-K-G judgment (e.g.^30–32^). The absence of the late frontal monitoring effect for typical items after categorical encoding might reflect the recruitment of different processes. This condition is associated with lower memory discrimination and much longer reaction times, suggesting relatively weak memory traces that may not provide the necessary information. Compared to other studies investigating recognition memory in NT adults, we only observed modest ERP old/new effects. This finding aligns with recent studies that provide evidence that familiarity-based recognition is associated with more variable and less robust ERP correlates^33,34^. We also provide evidence for a slightly more liberal response strategy in NT adults. Since ERP correlates are derived from all correct “old” responses, it is impossible to dissociate which precise trials were based on true recognition as opposed to correct guesses. Hence, ERP correlates of familiarity, in particular, are attenuated with a liberal response strategy^35^. Finally, the sequential presentation of the old/new and R-K-G judgments allowed us to disentangle both judgments, demonstrating that NTs activate previous knowledge for old/new judgments when they are not explicitly required to retrieve detailed item features. Therefore, they need to reinstate their memory in the subsequent step of R-K-G judgment. In previous source memory tasks^36,37^, NT participants have been shown to rely more on recollection than familiarity when a source memory judgment already includes the retrieval of source-specifying information. The sequential response setup likely led NT participants to bypass recollection for the old/new decision, possibly skewing the ERP patterns and underestimating the role of recollection.

In contrast, autistic participants exhibited slower reaction times and slightly lower recognition accuracy across all conditions^25^, while maintaining a neutral response criterion. Moreover, we do not see modulations in the early ERP time window in ASD adults, unlike those observed for the NT adults. Together, both results suggest that autistic individuals do not rely on familiarity-based responses. This finding aligns with the results of Massand and Bowler, who found an attenuated early frontal old/new effect associated with familiarity in ASD^26^. A group difference in the timing of retrieval processes was also observed in the second ERP time window, confirming that autistic adults rely on recollection-based processes. This finding supports the idea that individuals with ASD may have difficulty using familiarity as a reliable recognition cue, leading to a greater reliance on slower, more effortful recollection processes. In NT participants, we observed ERP differences primarily after the old/new response. This aligns with the idea that they reinstate item details in preparation for the R-K-G judgment. In contrast, autistic participants showed ERP effects already during the old/new decision phase, suggesting that they engaged recollection mechanisms earlier. This interpretation is further supported by the absence of significant ERP differences in the period preceding the R-K-G judgment, indicating that autistic individuals did not require additional feature reinstatement after making their initial recognition decision. Even though self-reported recollection rates were lower in autism, sequentially presenting both judgments was useful in clarifying that autistic adults are indeed able to engage in recollection-based processes; they only need more time. Future studies should consider using multiple task designs that contrast simultaneous with sequential old/new and R-K-G judgments to further this discussion.

While this study provides valuable insights into the neurocognitive processes underlying recognition memory in autistic and NT adults, a few limitations should be acknowledged. First, the focus on male participants limits the applicability of the results to female individuals with ASD, who may exhibit different memory processing patterns. Given the known sex differences in ASD prevalence, future research should target both male and female ASD participants to explore whether there are gender-related differences in recognition memory processes. Second, we acknowledge that a few trend-level results in the present study limit a conclusive interpretation of some aspects of our results. To uncover additional small magnitude effects, future studies should strive to recruit larger samples. Third, this study offers valuable insights into the complex interplay between episodic and semantic processes in memory retrieval. However, their individual contributions to memory processing in both autistic and NT individuals require further investigation. Finally, we must acknowledge the variable temporal dynamics of recognition memory retrieval, as participants may engage multiple cognitive processes either sequentially or simultaneously. This variability can lead to significant fluctuations in the timing and nature of neural responses, making it challenging to isolate specific processes within the ERP data at a group level. Consequently, the overlap of several cognitive processes could mask or distort the interpretation of ERP effects, particularly when different participants employ selective strategies for memory retrieval depending on the specific material and/or encoding context.

To conclude, this study provides evidence for distinct neurocognitive processes underlying recognition memory in individuals with and without ASD. Complementing self-report data, NT adults initially rely predominantly on familiarity, while autistic adults engage in more detailed and effortful recollection for old/new discrimination. This initial reliance on familiarity requires additional activation in the post-response period for the reinstatement of item details underlying subsequent R-K-G judgments for NT adults only. These differences are reflected in both behavioural discrimination and ERP patterns, suggesting that individuals with ASD may engage in different cognitive strategies to achieve comparable memory discrimination. These findings have important implications for designing interventions and support strategies tailored to the unique memory-processing needs of individuals with ASD. To enhance episodic retrieval in autistic participants, interventions should consider their alternative way to perform a judgment (criterion) in conjunction with processing time and cognitive processing styles. The use of specific instructions to probe either familiarity or recollection-based memory retrieval in both NT and ASD individuals may reveal whether the cognitive processes recruited in the present paradigm reflect a preference or a compensatory strategy in each group. Future research should continue to explore the interaction between semantic and episodic memory in ASD.

## Methods

### Participants

Fifteen male adults diagnosed with ASD and scoring above 70 points on the verbal subscale of the Wechsler Adult Intelligence Scale were matched with eighteen typically developed male participants in terms of age, education, and non-verbal general cognitive ability (for precise sample characteristics and diagnostic criteria see^25^). All participants were male, reported normal or corrected-to-normal vision, and had completed more than nine years of formal education. The study was conducted in accordancewith the Declaration of Helsinki and was approved by the ethical review board of the Faculty of Psychology at the University of Lisbon. All participants and their legal representatives were carefully informed of the participation conditions, and eachparticipant signed an informed consent form. The data from one NT adult was excluded from further analyses due to technical difficulties; one autistic adult was excluded due to extensive artifacts, and another autistic adult was excluded due to low memory discrimination performance. Thus, the data of 17 NT participants and 13 autistic participants were included in the current analyses (for sample characteristics, see Table 3).

**Table 3:** Demographic information of the samples.

		ASD	NT	Group differences
	N	13	17	
Age (years)	*M* (*SD*)	30.69 (6.03)	33.38 (9.51)	*t*(28)= −0.841, *p* =.41
Schooling (years)	*M* (*SD*)	14.54 (2.54)	15.17 (2.07)	*t*(28)=−0.749, *p* =.46
Non-verbal intelligence (Raven’s Standard Progressive Matrices, raw scores)	*M* (*SD*)	51.62 (6.53)	51.78 (4.87)	*t*(28)=−0.069, *p* =.95
Verbal competencies (Verbal subscale of Wechsler Adult Intelligence Scale, WAIS quotient)	QIV (*SD*)	108.69 (12.85)		
Diagnostic (Asperger’s Syndrome Diagnostic Scale, raw scores)	ASDS-ASD (*SD*)	102.92 (8.74)		

### Materials and procedure

The task consisted of a R-K-G paradigm with visual stimuli (500 × 500 pixels images) depicting common objects, varying in item typicality (typical vs. atypical) and encoding type (perceptual vs. categorical; see^14^). The encoding phase consisted of two tasks: one requiring more perceptual (visual complexity rating) encoding and the other more abstract (categorical sorting) encoding. Typicality was modulated implicitly, as an inherent property of categorical knowledge that reflects how representative an item is within its category^12^. In the *complexity rating task*, participants were asked to assess visual complexity by evaluating each image based on the number of traits, lines, and colors, using a 4-point scale. Due to the focus on the perceptual details of the image during the encoding phase, participants were expected to encode the item based on its perceptual details. In the *category sorting task*, participants indicated the best category to describe an item (i.e., inclusion alternative) in a 4-alternative choice paradigm (e.g., vehicles, mammals, fruits, furniture). This sorting task was expected to enhance the relevance of categorical schematic knowledge during encoding and maximize the predictive power of item-typicality in how to use, understand, and reason categories^12^. All participants engaged in both types of encoding tasks in a counterbalanced order, with a brief break (approximately 5 min) between tasks. During encoding, 160 images of common objects from eight different categories (i.e., birds, fruits, mammals, vegetables, vehicles, furniture, kitchen utensils, and clothes) were presented. The images were selected from normative studies of concepts and their related pictures conducted with Portuguese samples^36^ based on previous ratings for typicality (atypical: *M* = 4.75, *SD* = 0.01; typical: *M* = 6.39, *SD* = 0.03, *t*(158) = −16.14, *p* <.001, *d*_*z*_ = −1.280, CI 90% [1.10, 1.45]) and controlled for relevant dimensions in common objects’ processing such as arousal, valence, aesthetic appeal, and visual complexity (all *p*’s >.10^36,37^). Each encoding task included a set of items, equally distributed across four categories (counterbalanced across tasks) and representing two levels of item-typicality, with half of the items being typical, like a **robin** in the birds category, and half being atypical, such as a *penguin* in the birds category, within each category. Each item was presented once.

During a 20-minute retention interval (plus 5 min of instructions), participants performed the Raven’s standard progressive matrices. Hence, no similar stimulus material was used in this retention interval, nor were additional memory requirements imposed. Afterwards, participants performed the retrieval phase. This phase consisted of a yes-no recognition task, followed by subsequent phenomenological judgments. All encoded images (160 *old items*) were presented again, together with 106 *new images* of common objects. The new images followed the same criteria as the already encoded (old) images (all *p*s > 0.10). Participants saw an image (old or new item) and performed an old/new decision task (“Did you see the item?” - Yes/No). Whenever a ‘yes’ response was given, participants were asked to provide a phenomenological (R-K-G) judgment, indicating if they *Remember* (a recollective retrieval based on vivid details about the experience), *Know* (based on a sense of familiarity), or *Guess *(an uncertain feeling of having seen the item based on familiarity) having seen the item, in a forced-choice response option (e.g.^38,39^). At the end of the experiment, participants were thanked and debriefed.

### Electrophysiological recordings

For the EEG recording, we used 64 Ag/AgCl cap-mounted electrodes, plus two electrodes placed at the mastoids and four electrodes around the eyes, positioned on an extended 10–20 system^40^. The EEG was recorded using the BioSemi EEG System (BioSemi B.V., Amsterdam, the Netherlands). Data from all electrodes were recorded with an electrode offset within a 40 µV range. The electrode offset was generated at the junction of the skin and electrolyte solution under the EEG channels, which is a by-product of the direct current potentials, resulting in a voltage at the amplifier input^41^. We used four electrodes around the eyes (above and below the right eye, as well as at the left and right outer canthi) to record eye movements. The ground electrode was placed with the Common Mode Sense (CMS) active electrode and the driven right leg (DRL) passive electrode at the electrode positions PO1 and PO2, respectively, in the 10–20 system. The CMS was also used as an online reference. The sampling frequency was 2048 Hz.

### Data processing

We reduced the sampling frequency to 500 Hz. We used the spherical spline method^42^ for interpolating electrodes with many artifacts, as this method makes no assumption about the conductivity of the head tissues^43^ (with interpolation ranging from 0 to 5 electrodes per participant across both groups). The signal was re-referenced offline to the average of all cap-mounted electrodes (except for Iz, P10, and P9) using Brain Vision Analyzer 2.3 (Brain Products GmbH, Gilching, Germany). Since most of the relevant portions of ERPs in cognitive neuroscience consist of frequencies between 0.1 Hz and 40 Hz^24^ (at 48dB/oct), the EEG signal was filtered using a zero-phase shift Butterworth filter (the most commonly used filter^43^) as well as a 50 Hz notch filter. We corrected for eye movement artifacts using an independent component analysis. EEG segments were based on a time window of 500 ms before and 1500 ms after stimulus onset in the retrieval phase. Artifacts were removed semi-automatically when: (1) the amplitude difference between two sample points exceeded 50 µV, (2) the amplitude difference was more than 120 µV in an interval of 200 ms, or (3) a low amplitude of 0.5 µV occurred in a 100 ms interval. Due to artifact rejection, on average, 5.69% of all trials had to be removed in the NT group and 8.14% in the autistic group.

### Analyses

All analyses were performed with SPSS 29. Memory performance was defined by the sensitivity score Pr (Hit rate - False Alarm rate; cf^27^.), with scores ranging from 1 (perfect discrimination) to 0 (no discrimination). We also determined Br an as index for a potential response bias (FA/(1 - Pr), cf.^27^ specifying the rate of “old” responses given in the absence of a memory trace, with values of 0.5 indicating a neutral response criterion, while higher Br values indicate a tendency to guess “old” (i.e., liberal bias) and lower Br values indicate a tendency to guess “new” (i.e., conservative bias) when no memory trace can be retrieved. For these analyses, we computed a repeated-measures ANOVA on the within-subject factors Encoding type (Categorical vs. Perceptive) and Item Typicality (Typical vs. Atypical), as well as the between-subject factor Group (ASD vs. NT).

The EEG signal was segmented relative to the picture presentation during the yes-no recognition task in the retrieval phase and baseline**-**corrected using the 200 ms time window before stimulus onset (as recommended by Luck^24^). The signal was averaged per condition and participant only for items with correct responses. We evaluated the time windows 300–500 ms, 500–800 ms, and 900**–**1500 ms associated with familiarity, recollection, and memory retrieval monitoring, respectively. Based on previous findings, we investigated the mean amplitude in the time windows as mean activity of the electrode sites F7, F5, FT7, FC5, and C5 (left fronto-central ROI to assess familiarity); POz, PO4, PO3, Oz, O1 and O2 (parieto-occipital ROI to assess recollection); and Fpz, Fp1, and AF7 (left prefrontal ROI to assess retrieval monitoring), respectively. The ERP analyses were performed separately for each group, as statistical interactions of electrophysiological data between groups of participants may reflect anatomical differences rather than distinct cognitive processes employed in each group (especially when comparing clinical and NT samples; for a more detailed explanation, see^23^). Since we predicted selective effects only for some conditions, interaction effects were followed up at a trend level (*p* <.10), but only significant results of the planned contrasts are reported for the sake of brevity. The Greenhouse-Geisser correction was applied when the assumption of sphericity was violated.

## Data Availability

Upon data collection, we did not receive permission to make the raw EEG and behavioural data of our participants with and without a diagnosis of ASD publicly available. Hence, we cannot upload raw data for the present investigation into a repository. The datasets used and/or analysed for the purpose of the present investigation are available from the corresponding author on reasonable scientific request.
